# Anomaly Detection of Acoustic Signals in Ultra-High Voltage Converter Valves Based on the FAVAE-AS

**DOI:** 10.3390/s25154716

**Published:** 2025-07-31

**Authors:** Shuyan Pan, Mingzhu Tang, Na Li, Jiawen Zuo, Xingpeng Zhou

**Affiliations:** 1 School of Electrical and Information Engineering, Changsha University of Science & Technology, Changsha 410114, China; 202205020312@stu.csust.edu.cn; 2 School of Energy and Power Engineering, Changsha University of Science & Technology, Changsha 410114, China; 22106011225@csust.edu.cn; 3 School of Economics & Management, Changsha University of Science & Technology, Changsha 410114, China; lina@stu.csust.edu.cn; 4 School of Computer Science and Technology, Changsha University of Science & Technology, Changsha 410114, China; cyclonezxp@stu.csust.edu.cn

**Keywords:** converter valve fault diagnosis, convolutional neural networks, long short-term memory networks, intelligent operation and maintenance

## Abstract

The converter valve is the core component of the ultra-high voltage direct current (UHVDC) transmission system, and its fault detection is very important to ensure the safe and stable operation of the transmission system. However, the voiceprint signals collected by converter stations under complex operating conditions are often affected by background noise, spikes, and nonlinear interference. Traditional methods make it difficult to achieve high-precision detection due to the lack of feature extraction ability and poor noise robustness. This paper proposes a fault-aware variational self-encoder model (FAVAE-AS) based on a weak correlation between attention and self-supervised learning. It extracts probability features via a conditional variational autoencoder, strengthens feature representation using multi-layer convolution and residual connections, and introduces a weak correlation attention mechanism to capture global time point relationships. A self-supervised learning module with six signal transformations improves generalization, while KL divergence-based correlation inconsistency quantization with dynamic thresholds enables accurate anomaly detection. Experiments show that FAVAE-AS achieves 0.925 accuracy in fault detection, which is 5% higher than previous methods, and has strong robustness. This research provides critical technical support for UHVDC system safety by addressing converter valve acoustic anomaly detection. It proposes an extensible framework for industrial intelligent maintenance.

## 1. Introduction

As the core component of the UHVDC transmission system, the operation state of the converter valve is directly related to the security and stability of the power system. With the increasing scale and complexity of power systems, the fault detection and diagnosis of converter valves face higher requirements. Voiceprint signal has become an important means of fault detection because of its non-invasive, real-time, and rich information dimensions. However, due to the widespread background noise, nonlinear interference, and the diversity of fault modes in the industrial environment, the traditional fault detection methods often make it challenging to achieve unity of high accuracy and robustness.

Early research focused on physical modeling, signal analysis, and traditional machine learning methods. Gu A et al. [1] proposed a simplified modeling method based on selective filters for fault detection. Although this method achieved high detection accuracy, it had high requirements for system modeling and was not suitable for complex practical application scenarios. Vasileios Psaras et al. [2] proposed a new adaptive threshold protection method based on non-unit WT, which improved the detection ability in a high-noise environment but still failed to capture the nonlinear characteristics of the signal. Bansal M et al. [3] proposed a complete protection scheme for ultra-high voltage converter valve fault detection, classification, and location based on support vector machines (SVMs). Although this method improves the diagnosis accuracy to a certain extent, it relies on artificial feature extraction, which is complex, prone to losing key information, and makes it challenging to deal with complex and changeable fault modes.

Traditional fault diagnosis methods rely on expert experience and artificial feature extraction, which are inefficient, have poor adaptability to complex operating conditions, and make it challenging to meet the stringent requirements of modern industrial systems for real time and robustness. In contrast, deep learning technology, with its end-to-end feature learning ability, provides a new idea for fault detection. Convolutional neural networks (CNNs) are one of the first deep learning models applied to fault detection tasks because of their excellent local feature extraction ability. Dharma et al. [4] proposed a fault detection method based on empirical wavelet transform (EWT), multi-scale fuzzy entropy (MSFE), and a whale optimization algorithm convolutional neural network. However, its local modeling characteristics limited the modeling ability of the medium- and long-term dependence of the signal, and its performance was insufficient in processing the dynamic evolution of time-series signals. In addition, CNNs are highly sensitive to nonlinear noise in complex industrial environments, and it is difficult to achieve the robust extraction of key fault features.

In order to make up for the deficiency of CNNs in time dimension modeling, recurrent neural networks (RNNs) [5] and their improved model, long and short-term memory networks (LSTMs) [6], are widely introduced into fault detection tasks. These models can capture the dynamic dependence of signals in the time dimension through a recursive structure and a gating mechanism. Nguyen-Da [7] and others proposed a real-time intelligent AIoT system based on a convolutional neural network and an extended short-term memory network (CNN-LSTM). This method effectively captures the signal changes with different time steps and assists in fault feature extraction. However, the ability of local feature modeling in the signal space dimension is limited, and it is difficult to extract the key local fault modes fully. In addition, the robustness of LSTMs to peak noise and nonlinear interference is poor, and it is easily affected by the background noise in a complex transmission environment, which reduces the fault detection performance. Although the simplified modeling method based on selective filtering proposed by Khodarahmi M et al. [8] achieves high detection accuracy, it has high requirements for system modeling and is not suitable for complex practical application scenarios. The adaptive threshold protection method based on wavelet transform proposed by Marvasti et al. [9] improves the detection ability in a strong noise environment but still lacks sufficient nonlinear signal feature capture ability. Yang H and others [10] proposed a high-voltage DC fault detection, classification, and location scheme based on SVMs to improve diagnosis accuracy. However, due to the high dependence on artificial feature extraction, the feature extraction process is complex, and it is easy to lose key information, which makes it challenging to deal with complex and changeable fault patterns.

The existing deep learning model still faces four significant challenges in processing the voiceprint signal of the converter valve: First, the traditional model mainly focuses on local feature extraction, which makes it challenging to take into account the local details and global time dependence in the signal at the same time. Secondly, limited training samples and complex noise environment may lead to overfitting of the model. Third, the existing models lack effective modeling of uncertainty in the process of signal reconstruction. Fourth, the lack of an effective anomaly quantification mechanism makes it difficult to identify and interpret anomaly patterns accurately. To solve the above problems, this paper proposes a fault-aware variational automatic encoder model based on weak correlation attention and self-supervised learning (FAVAE-AS), which aims to overcome the limitations of existing methods in the fault detection of the converter valve voiceprint signal. Compared with the existing technology, the FAVAE-AS model has the following innovations:

**(1)**  Probabilistic feature extraction framework: Aiming at the problem of the insufficient modeling of probability distribution caused by the deterministic feature representation of traditional models, FAVAE-AS constructs probabilistic feature space based on the conditional variation autoencoder (CVAE), extracts multi-scale local features through multi-layer convolution, and introduces residual jump connection to optimize the gradient propagation path. This method not only enhances the ability of feature expression but also effectively solves the problem of gradient disappearance in deep network training.
**(2)**  Global local collaborative weak correlation attention mechanism: in order to solve the shortage of CNN/LSTM long-time-series modeling ability, FAVAE-AS designed the global correlation weight calculation module, combined with gating attention and sparse constraints, and adaptively focused on the low correlation mode of abnormal areas to effectively capture the weak correlation mode at abnormal points, significantly improving the modeling ability of the model for complex time-series signals.
**(3)**  Noise robust self-supervised learning enhancement module: for the generalization bottleneck in limited samples and a complex noise environment, FAVAE-AS is integrated into the self-supervised learning enhancement module. Through six fundamental signal transformation and combination strategies, the module significantly improves the generalization performance of the model and alleviates the overfitting phenomenon.
**(4)**  Interpretable dynamic anomaly quantification system: In order to break through the limitations of traditional fixed thresholds and manual analysis, FAVAE-AS proposed the correlation inconsistency quantification method of Kullback–Leibler (KL) divergence, and combined with the dynamic threshold strategy, realized the accurate identification and reliable evaluation of anomalies. This method not only improves the accuracy of anomaly detection but also enhances the interpretability of detection results.

## 2. Related Work

### 2.1. Feature Extraction and Error Reconstruction

In the field of time-series anomaly detection, feature representation learning and reconstruction error analysis have formed a multi-paradigm technology system. Traditional dimensionality reduction methods such as principal component analysis (PCA) [11] and independent component analysis (ICA) [12] construct low-dimensional representations through linear transformation, but their representation of nonlinear patterns is increasingly prominent in complex industrial voiceprint analysis. The introduction of deep learning technology has promoted a paradigm breakthrough in this field. Zhang K [13] took the lead in introducing the variational autoencoder (VAE) architecture into feature extraction. The CVAE proposed by Lee M [14] realizes the decoupling characterization of feature space through the conditional guidance mechanism. The β-VAE variant of Burgess C [15] achieves a balance between feature capacity control and dimensional decoupling through superparametric adjustment.

For the field of mechanical fault diagnosis, Chen D [16] designed a convolutional VAE network to extract bearing fault features, and Xu X [17] built a hierarchical VAE architecture to capture multi-scale time–frequency features. However, the existing methods of multi-focus static feature extraction are insufficient to model the long-range time dependence contained in the converter valve voiceprint signal. Recent studies have begun to focus on the uncertainty modeling of representational learning. Durasov N [18] proposed an uncertainty estimation method based on Monte Carlo dropout, and Diekmann O [19] built a dual-channel uncertainty framework to distinguish random noise and cognitive uncertainty. In terms of reconstruction error measurement, the weighted reconstruction loss of Hsu Y [20] and the adaptive threshold mechanism of Cao L [21] show that the traditional mean square error (MSE) index has difficulty meeting the needs of complex industrial scenarios. Despite these advances, the existing methods still have limitations in solving the unique challenges of the voiceprint signal of the converter valve, which promotes the development of methods that integrate new attention mechanisms and self-monitoring learning strategies.

### 2.2. Self-Supervised Mechanisms Based on Deep Learning

As an emerging technology in the field of deep learning, self-supervised learning has made significant progress in feature representation learning in recent years. The comparative learning framework, similarly proposed by Yeh C et al. [22], learns robust feature representation by maximizing mutual information between samples, while the Moco framework introduced by Sowrirajan H et al. [23] improves the efficiency of comparative learning through a momentum encoder and a queue mechanism. In time-series analysis, Shurrab S et al. [24] developed a self-supervised learning method based on time prediction, which proved the importance of self-supervised tasks in improving the generalization ability of the model.

For industrial signal processing, Xu J et al. [25] proposed a multi-view self-supervised learning framework, which constructed auxiliary tasks through various signal transformations, significantly improving the accuracy of fault diagnosis. In terms of data enhancement strategy, Pan C et al. [26] systematically studied the impact of various signal transformation methods on the performance of the model and proposed an adaptive enhancement strategy selection mechanism. Chen Z et al. [27] explored the signal enhancement method based on physical prior knowledge and proved the importance of domain knowledge in improving the effectiveness of self-supervised learning.

In the scene of converter valve voiceprint analysis, the existing methods face two technical bottlenecks: one is how to design an enhancement strategy that matches the physical characteristics of the signal, and the other is how to balance self-supervised learning and the main task. These challenges prompt us to propose a self-supervised learning enhancement module that integrates multiple signal transformations and dynamic weight adjustment to improve the ability of the model to detect abnormal patterns.

### 2.3. Associative Attention Mechanism for Industrial Signals

The attention mechanism has been widely used in deep learning and has achieved remarkable success. The transformer architecture proposed by Ding Y et al. [28] innovated sequence modeling through the self-attention mechanism, laying the foundation for subsequent research. In time-series analysis, the informer model proposed by Li Y et al. [29] effectively solves the problem of long-series modeling through the sparse attention mechanism. At the same time, the log transformer of Huang S et al. [30] designed a hierarchical attention structure for system log analysis. In the field of industrial signal processing, Yan J et al. [31] proposed a multi-head spatiotemporal attention network, which improved the accuracy of fault diagnosis by capturing the multi-scale time dependence. Rudin C et al. [32] explored the attention mechanism guided by physical knowledge and enhanced the interpretability of the model by introducing an a priori constraint. For anomaly detection tasks, Song C et al. [33] developed a comparative attention framework to improve detection performance by learning the attention difference between normal and abnormal patterns. The weak correlation attention mechanism (WAA) proposed in this article differs from traditional self-attention in that its core lies in focusing on low-correlation patterns in anomalous regions through sparse constraints. In standard signals, the weights associated with time points are evenly distributed. Abnormal points have significantly reduced correlation weights with other points due to feature mutations, and WAA achieves anomaly localization by modeling this weak correlation.

### 2.4. Inconsistency Quantification for Anomaly Detection

**Figure 1:**
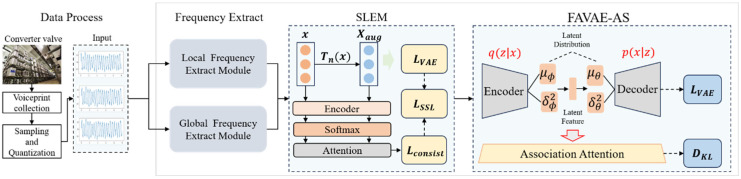
FAVAE-AS model architecture.

The inconsistency quantification method in anomaly detection has always been a research hotspot. Qian Q et al. [34] first proposed an inconsistency measurement method based on information entropy, which identifies anomalies by calculating the difference between the sample distribution and the reference distribution. In time-series analysis, Niennattrakul V et al. [35] developed a hybrid measurement method combining dynamic time warping (DTW) distance and KL divergence, which significantly improved the accuracy of anomaly detection. Grant J et al. [36] explored a framework for quantifying inconsistency based on physical model constraints, which improved the interpretability of test results by incorporating prior knowledge. In the field of industrial signal processing, Klein R et al. [37] proposed an adaptive multi-scale inconsistency measurement method, which enhanced the robustness of detection by considering the multi-layer characteristics of the signal. In terms of threshold setting, Sun Q et al. [38] developed a dynamic threshold adjustment strategy based on statistical learning, which effectively solved the problem of threshold selection in non-stationary signal detection. However, the existing methods still face two significant challenges in processing the converter valve voiceprint signal: one is how to effectively integrate the reconstruction error and the inconsistent information of correlation features; the other is how to design a reasonable dynamic threshold strategy to adapt to the non-stationary characteristics of the signal. These challenges prompt us to propose a correlation inconsistency quantification method based on KL divergence, combined with an adaptive threshold strategy, to improve the accuracy and reliability of anomaly detection. The overall structure of the FAVAE-AS model, including the feature extraction module, self-supervised learning enhancement module, association attention mechanism, and anomaly detection module, is shown in Figure 1. In Figure 1, the ‘weakly correlated attention module (WAA)’ calculates the global time point association weight matrix A, combined with a sparse constraint, adaptively focusing on low correlation patterns in anomalous regions.

Figure 1 presents the overall structure of the FAVAE-AS model, which includes the feature extraction module, self-supervised learning enhancement module, association attention mechanism, and anomaly detection module.

## 3. Method

In order to solve the fault detection challenges caused by background noise and nonlinear interference in the voiceprint signal of the converter valve, a fault-aware variational automatic encoder model with correlated attention and self-supervised learning (FAVAE-AS) is proposed in this paper. Based on the existing fully connected variational automatic encoder (FAVAE), the model innovatively integrates the weak association attention mechanism (WAA) and self-supervised learning enhancement module (SLEM). Through multidimensional feature learning, outlier correlation analysis, inconsistency quantification, and self-monitoring feature enhancement, the model improves the accuracy and generalization performance of fault detection.

**Figure 2:**
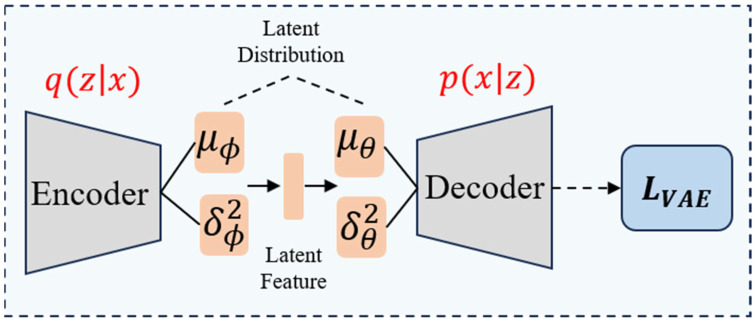
The probabilistic feature extraction framework structure.

**Figure 3:**
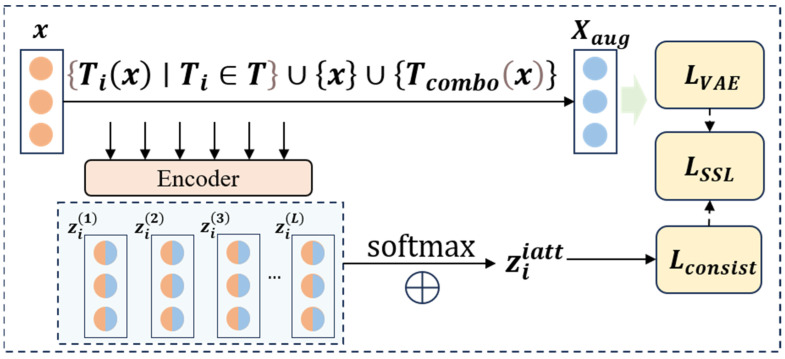
The self-supervised learning enhancement structure.

**Figure 4:**
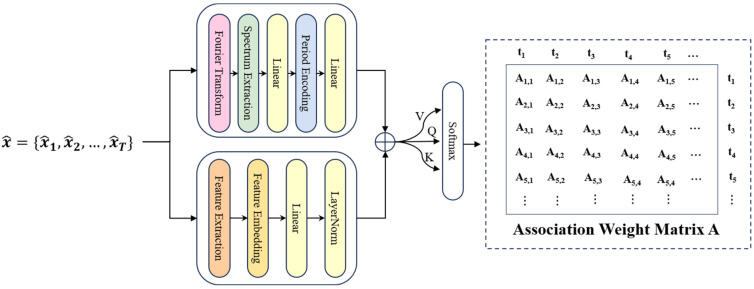
The weak association attention mechanism structure.

The overall structure of the FAVAE-AS model is divided into four main modules (see Figure 1 for the overall architecture): first, the encoder-decoder structure (Formulas (1) and (2)) based on FAVAE extracts the multi-scale potential features of the signal through multi-layer convolution and residual jump connection (Figure 2), and the uncertainty is modeled by variational reasoning(Formulas (3) and (4)) in order to suppress the feature drifting under noise interference; Second, a self-supervised enhancement module (Figure 3) is introduced to generate adversarial samples using six physically-inspired signal transformations (Formulas (7)–(13)); To optimize the feature consistency constraint (Formula (20)) and improve the generalization ability under limited labeled data, all modules optimize the objective function (Formula (21)) through end-to-end joint training, which incorporates a dynamic weight adjustment mechanism (Formula (22)); furthermore, the weakly-associated attention module (Formulas (23) and (24)) calculates the global time-point association weights, and utilizes sparse-attention low-association patterns focusing on the anomalous region (Figure 4) to solve the traditional CNN/LSTM’s long temporal dependence modeling defects; finally, based on the KL scattering metric to measure the association inconsistency (Formula (26)), combined with the sliding window smoothing (Formula (27)) and adaptive thresholding strategy (Formula (28)), dynamically generating the interpretable anomaly scores (Formula (29)) to achieve the precise localization of the faulty signals and the visual analysis. Each module optimizes the objective function through end-to-end joint training, and improves the detection F1-score by 5% compared to the baseline model under the fusion of reconstruction error (Formula (6)) and correlation variance score decision, significantly enhancing the detection robustness in complex industrial scenarios detection robustness in complex industrial scenarios.

### 3.1. Probabilistic Feature Extraction Framework

Feature extraction is one of the core steps of the FAVAE-AS model. The main goal is to mine potential features from the voiceprint signal of the converter valve through a conditional variation automatic encoder (FAVAE) and use these features to identify abnormal patterns in the signal. The key to this process is to convert the high-dimensional time-series signal into a low-dimensional potential spatial representation while ensuring that the potential representation retains the key features of the signal as much as possible. Feature extraction provides basic support for the performance improvement of the whole model and is a key step in the process of signal anomaly detection.

In practical applications, the voiceprint signal of the converter valve usually contains a lot of redundant information and noise interference. In order to extract the most representative features from the signal, this paper uses a conditional variational automatic encoder structure based on deep learning. This structure can not only effectively reduce the dimension of the signal but also capture the internal distribution characteristics of the signal through probability modeling, providing a more reliable feature representation for subsequent anomaly detection.

For a given time-series signal $x=\{x_{1},x_{2},\ldots ,x_{t}\}$, the encoder maps the signal to the potential spatial feature Z through nonlinear transformation. In order to enhance the ability of feature expression, the encoder uses a multi-layer convolutional neural network structure to extract the local features of the signal layer by layer and reduce the dimension. The conditional probability distribution of potential space is as follows:

(1) $$q_{\phi }(z|x)=N(z;\mu_{\phi }(x),diag(\sigma_{\phi }^{2}(x)))$$

where $\mu_{\phi }(x)$ and $\sigma_{\phi }^{2}(x)$ are the mean and variance of the potential space, which are learned from the encoder parameter ϕ. This probabilistic modeling method enables the model to better handle the uncertainty in the signal and improve the robustness of feature extraction.

After feature extraction, the decoder uses the potential feature Z to reconstruct the original signal, trying to recover the main features of the time series. The decoder also adopts a symmetrical convolutional neural network structure and uses a transpose convolution operation to gradually restore the time dimension of the signal. The conditional probability distribution of decoding is as follows:

(2) $$p_{\theta }(x|z)=N(x;\mu_{\theta }(z),diag(\sigma_{\theta }^{2}(z)))$$

where $\mu_{\theta }(z)$ and $\sigma_{\theta }^{2}(z)$ are the outputs of the decoder, and the parameters are learned from θ. By introducing the probability decoder, the model can better deal with the uncertainty in the reconstruction process so as to improve the quality of reconstruction.

In order to optimize the model parameters, the variational automatic encoder (VAE) uses the training target based on the lower bound of evidence (ELBO). According to Bayesian inference, the logarithmic likelihood of input signal x can be decomposed into

(3) $$logp_{\theta }(x)=log\int_{z}\;p_{\theta }(x|z)p(z)dz$$

Due to the difficulty in directly calculating the posterior $p_{\theta }(z|x)$, introducing the variational distribution $q_{\phi }(z|x)$, and applying Jensen’s inequality, the lower bound of logarithmic likelihood can be obtained:

(4) $$log\;p_{\theta }(x)\geq E_{q_{\phi }(z|x)}\left[logp_{\theta }(x|z)\right]-KL\left(q_{\phi }(z|x)∥p(z)\right)$$

The lower bound is ELBO, and by maximizing ELBO, the logarithmic likelihood can be approximately optimized. Among them, p(z)=N(0,I) is the standard normal prior, and the KL divergence constraint ensures that the potential distribution has good generalization and avoids the problem of feature collapse.

(5) $$L_{VAE}=E_{q_{\phi }(z|x)}[logp_{\theta }(x|z)]-KL(q_{\phi }(z|x)∥p(z))$$

where p(z)=N(0,I) is the standard normal distribution and KL is the Kullback–Leibler divergence. The goal is to make the feature distribution in the potential space close to the standard normal distribution and ensure the universality and robustness of the feature. In the training process, the model uses the random gradient descent algorithm to optimize the lower bound of evidence (ELBO) objective function and constantly adjusts the encoder and decoder parameters so that the model can learn the essential characteristics of the signal. In order to improve the stability of training, an Adam optimizer is used, and a learning rate attenuation strategy is introduced. In addition, regularization techniques such as dropout are used to prevent overfitting during training. By optimizing the above objective function, the model can not only extract stable potential features but also evaluate the abnormal degree of the signal by reconstruction error $\varepsilon_{rec}$:

(6) $$\varepsilon_{rec}=∥x-\hat{x}\;∥$$

If the value of the reconstruction error $\varepsilon_{rec}$ is too large, it usually indicates that the signal may contain abnormal features, which provides a preliminary basis for subsequent correlation feature analysis. In order to enhance the reliability of the reconstruction error, the combination of mean square error (MSE) and mean absolute error (MAE) is used to measure the reconstruction quality of the signal more comprehensively. In addition, in order to improve the effect of feature extraction, the residual connection structure is introduced into the encoder and decoder. This jump connection mechanism effectively alleviates the gradient disappearance problem in deep network training, while retaining more original signal information.

### 3.2. Self-Supervised Learning Enhancement

In order to further improve the generalization ability of the model and avoid overfitting, this paper designs a self-supervised learning enhancement module (SLEM). In the actual voiceprint signal analysis of the transfer valve, due to the limited number of standard samples and the scarcity of abnormal samples, the model is often overfitted, resulting in poor detection performance of the invisible abnormal mode. To solve this problem, the SLEM constructs a rich set of self-supervised learning tasks to guide the model to learn the intrinsic features and invariants of the signal so as to enhance the robustness and generalization ability of feature extraction. The structure of the self-supervised learning enhancement module is shown in Figure 3. Adversarial samples are generated through six types of signal transformations, and the dynamic weight mechanism (Formula (20)) balances the training weights of the main task and the self-supervised task.

In the self-supervised learning framework, the design of the data enhancement strategy directly affects the learning effect of the model. Based on the physical characteristics and prior knowledge of the voiceprint signal of the conversion valve, a set of signal conversion functions is designed in this paper. Specifically, we propose six basic transformation operations: Gaussian noise injection, time reversal, time window replacement, amplitude scaling, signal reversal, and Savitzky–Golay smoothing. Gaussian noise injection transform is defined as

(7) $$T_{1}(x)=x+\epsilon ,\epsilon ∼N(0,\sigma^{2})$$

The transformation adds Gaussian white noise with a mean value of 0 and variance $\sigma^{2}$ to the original signal XXX. This operation enhances the robustness of the model to environmental noise. Time reversal transformation is defined as

(8) $$T_{2}\left(xt\right)=x_{T-t+1},\;\;\;\;t\in [1,T]$$

This conversion reverses the order of the time series, where t is the time index and TTT is the total length of the series. This operation helps the model learn the time symmetry of the signal. The time window displacement transformation is defined as

(9) $$T_{3}(x)=Concat({\left\{x_{wi}\right\}}_{i=1}^{k},\;\;\;\;w_{i}∼Perm([1,k])$$

The formula describes the process of dividing the signal into k windows and arranging them randomly. In order to maintain a smooth transition at the window boundary, a smooth transition function is introduced. The smooth transition function is defined as

(10) $$x_{smooth(t)}=\alpha (t)xw_{i}(t)+(1-\alpha (t))xw_{i+1}(t)$$

where α(t) is the weight function of the smooth transition at the window boundary to ensure smooth continuity between adjacent windows. The amplitude scaling transform is defined as

(11) $$T_{4}(x)=\alpha x,\alpha ∼U(a,b)$$

The transform uses a random scaling factor uniformly sampled from the interval [a, b] to adjust the amplitude of the signal. This operation simulates natural changes in signal strength. The inverse signal transform is defined as

(12) $$T_{5}(x)=-x$$

This is a polarity reversal operation, which is helpful for the model to understand the positive and negative symmetry of the signal. Savitzky–Golay smooth transform is defined as

(13) $$T_{6}(x)=SG\left(x,w,p\right)$$

The transform applies Savitzky–Golay filtering to smooth the signal, where WWW is the filter window length and P is the order of the fitting polynomial. To further enrich the conversion types, this paper introduces a conversion combination operation:

(14) $$T_{combo}\left(x\right)=T_{i}\left(T_{j}\left(x\right)\right),\;\;\;\;i,j\in \left\{1,\ldots ,6\right\},\;\;\;\;i\neq j$$

This formula describes the process of applying two different basic transformations to the signal sequence, resulting in more complex transformation effects. Then, we build an enhanced sample set based on the above transformation:

(15) $$X_{aug}=\{T_{i}(x)|T_{i}\in T\}\cup \{x\}\cup \{T_{combo}\left(x\right)\}$$

The set includes the original signal, all signals transformed by the basic transformation, and signals transformed by the transformation combination. For each enhancement sample, we use a multi-layer encoder to extract features:

(16) $$z_{i}^{\left(l\right)}=E_{\phi }^{\left(l\right)}\left(T_{i}\left(x\right)\right),\;\;\;\;l\in \{1,\ldots ,L\}$$

This formula represents the feature extraction process of the first layer encoder for the transformed signal and generates a multi-scale feature representation. To effectively use multi-layer features, we introduce an attention mechanism:

(17) $$\alpha_{l}=softmax(W_{a}z_{i}^{\left(l\right)}+b_{a})$$

(18) $$z_{i}^{iatt}=\sum_{i=1}^{L}\alpha_{l}\;z_{i}^{\left(l\right)}$$

These two formulas describe the process of calculating attention weights and aggregating weighted features, where the sum is a learnable parameter. The loss function of self-supervised learning consists of two parts:

(19) $$L_{SSL}=\frac{-1}{N}\sum_{i=1}^{N}\sum_{k=0}^{6}y_{i},klog({\hat{y}}_{i,k})+\beta L_{consist}$$

where $y_{i,k}$ represents the classification target label of the i-th sample after the k-th signal transformation, which is used to guide the model to learn the invariance of transformed features. ${\overset{ˆ}{y}}_{i,k}$ is the predicted probability distribution of the transformed sample by the model, and the classification task is optimized through cross-entropy loss.

The first is the cross-entropy loss, which is used to optimize the transformation classification task; the second is the loss of feature consistency:

(20) $$L_{consist}=\frac{1}{N}\sum_{i=1}^{N}{∥z_{i}-z_{i}^{aug}∥}_{2}^{2}$$

This loss ensures that the feature representation under similar transformations is consistent. The final total loss function is

(21) $$L_{otal}=L_{VAE}+\lambda (t)L_{SSL}$$

The dynamic weight λ(t) changes with time:

(22) $$\lambda (t)=\lambda_{0}\cdot exp(-\gamma t/T)$$

This dynamic weight adjustment mechanism ensures that the model can reasonably balance the reconstruction task and the self-supervised learning task in the training process.

### 3.3. Weak Association Attention Mechanism

The weak association attention (WAA) mechanism calculates the global correlation weights of time points in the signal. It combines sparse constraints to focus on low correlation patterns in anomalous regions adaptively.

The traditional variational autoencoder (VAE) and convolutional neural network (CNN) methods mainly focus on local feature extraction, which makes it challenging to capture global correlation in time-series data. In order to overcome this limitation, this paper designs a weak association attention (WAA) mechanism, which calculates the global association weight between time points in the signal to identify the weak association pattern of abnormal points.

In time-series signals, outliers usually lead to significant changes in signal characteristics and may affect subsequent time points. Therefore, the correlation weight between abnormal points and other time points is usually much lower than that of regular points. By modeling these global associations, the WAA mechanism can effectively identify abnormal patterns.

The proposed associative attention mechanism is inspired by the self-attention structure in the transformer, but many of them are especially suitable for the characteristics of the voiceprint signal of the converter valve. First, the reconstructed signal $\hat{x}=\{{\hat{x}}_{1},{\hat{x}}_{2},\ldots ,{\hat{x}}_{T}\}$ is linearly transformed to generate a query, keyword, and value matrix Q,K,V:

(23) $$Q=W_{Q}\hat{x},\;\;\;\;K=W_{K}\hat{x},\;\;\;\;V=W_{V}\hat{x}$$

where $W_{Q}$, $W_{K}$, and $W_{V}$ are learnable weight matrices. These parameters are learned during training. In order to enhance the feature representation, nonlinear activation functions (such as ReLU) and normalization operations are introduced after each linear transformation. The design not only improves the nonlinear modeling ability of the model but also stabilizes the training process. The correlation weight matrix A is calculated by using the self-attention mechanism:

(24) $$A=softmax(\frac{QK^{⊤}}{\sqrt{d}})$$

where D is the dimension of potential characteristics. The association weight matrix captures the global relationship between time points in the time series. In the normal mode of the signal, the correlation between time points is usually uniform. In contrast, in the abnormal mode, the correlation weight between the abnormal point and other time points is significantly lower. This weak correlation pattern is reflected in the weight distribution of a, which is used as the input of subsequent quantization modules. In the correlation weight matrix A, the row vectors of outliers show a significant increase in KL divergence with the standard normal prior, indicating a ‘weak correlation’ feature.

### 3.4. Association Inconsistency Quantification

In order to distinguish abnormal points from normal points more accurately, this paper proposes a quantitative method of correlation inconsistency, which directly quantifies the abnormal characteristics of each time point. This method not only considers the strength of correlation between time points but also introduces constraints based on physical prior knowledge to improve the accuracy and interpretability of anomaly detection. Firstly, based on the relative position of time points, a priori incidence matrix P is defined to characterize the theoretical correlation strength between time points. Considering the physical characteristics of the voiceprint signal of the transfer valve, the elements of the prior incidence matrix are defined as

(25) $$P_{ij}=exp(-|i-j|/\sigma )$$

where σ is a superparameter that controls the rate of correlation decay. This parameter should be adjusted according to specific application scenarios. Then the Kullback–Leibler (KL) divergence between the self-attention weight matrix A and the prior incidence matrix P is calculated to quantify the correlation inconsistency at each time point.

(26) $$D_{KL}=\sum_{i=1}^{T}\sum_{j=1}^{T}A_{ij}log\frac{A_{ij}}{P_{ij}}$$

A large $D_{KL}$ value indicates that the time point deviates from the normal mode, indicating that this may be abnormal. To improve the stability of quantization results, moving window averaging and exponential smoothing techniques are also used in this paper.

(27) $$D_{KL}^{S}KLs(t)=\alpha D_{KL}(t)+(1-\alpha )D_{KL}^{S}(t-1)$$

where α is the smoothing factor. The processing effectively reduces the impact of noise and improves the reliability of anomaly detection. Finally, set the adaptive threshold to determine the exception:

(28) Threshold(t)=μ(t)+βσ(t)

where μ(t) and σ(t) are the mean and standard deviation of the local time window, and β\beta β is the sensitivity parameter. This dynamic threshold strategy can better adapt to the non-stationary characteristics of the signal and improve the detection accuracy. Finally, the model combines the reconstruction error $\varepsilon_{rec}$ and the correlation inconsistency score $D_{KL}$ to calculate the abnormal score at each time point.

(29) $$S_{anomaly}=\lambda \varepsilon_{rec}+\beta D_{KL},$$

where λ and β are the weight coefficients controlling the influence of each part on the abnormal score. If the abnormal score $S_{anomaly}$ exceeds the set threshold, the time point is classified as abnormal.

## 4. Experiments and Analyses

### 4.1. Data Collection

In this paper, the acoustic pattern sensor collects the acoustic pattern signal generated by the conversion valve. The acoustic amplifier first amplifies the collected acoustic signals and then continuously collects them by the data acquisition card. Finally, the data are uploaded to the server through a switch.

**Figure 5:**
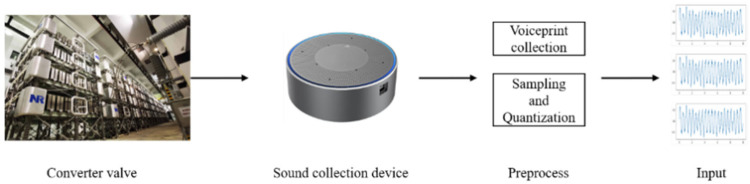
Voiceprint data processing process.

The experiment uses a model, with a sampling rate of 48 kHz, which is a high-precision acoustic ripple sensor, to collect the acoustic ripple signals generated during the operation of the converter valve. The AD620 amplifier (Analog Devices Inc, Norwood, MA, USA) gain adjusts the raw signals, then continuously records by the NI-PXIe-6368 (Emerson, St. Louis, MI, USA) data acquisition card at a sampling frequency of 10 kHz, and finally uploads to the data center through a Gigabit Ethernet switch. Figure 5 shows the complete sound data processing flow, including three core steps of signal preprocessing, segmentation, and z-score normalization, to ensure that the data quality meets the requirements of the model inputs, as shown in the flow below.

### 4.2. Model Parameters and Training Environment

All experiments were conducted on the Linux operating system, and the overall algorithmic framework was based on the PyTorch deep learning environment. The specific configuration of the experimental environment is shown in Table 1, and the experimental parameter settings for the FAVAE-AS model are detailed in Table 2. The model was trained for a total of 50 iterations, with the learning rate set to 0.00001. The maximum sequence length of the input data was limited to 512 tokens to ensure efficient processing while maintaining contextual information. A batch size of 32 was used to balance computational efficiency and training stability. To prevent overfitting, a dropout rate of 0.3 was used during training. The AdamW optimizer was chosen because of its ability to efficiently adapt to different parameter learning rates and its ability to decouple the weight decay from the gradient update process. These parameter settings were chosen based on empirical tuning to optimize model performance and convergence.

### 4.3. Results Analysis

To verify the robustness of the model in complex noise environments, the experiments use the Noisex-92 noise library (containing three types of noise, including mechanical noise, pink noise, etc.) to construct the test set, in which the pink noise is linear noise, and the remaining two types of noise are nonlinear noise. Accuracy calculation formula:

(30) $$Accuracy=\frac{TP+TN}{TP+TN+FP+FN}$$

where TP (True Positive) is the number of correctly detected fault samples, TN (True Negative) is the number of correctly detected standard samples, FP (False Positive) is the number of incorrectly detected standard samples, and FN (False Negative) is the number of missed fault samples.

By setting a total of five signal-to-noise ratios (SNRs) from 3 dB to 18 dB, the noise is injected into the original acoustic pattern signal of the converter valve, and the detection performances of the SPOT [39], SRCNN [40], Anomaly Transformer, FAVAE, and FAVAE-AS models are comparatively analyzed, and the specific results are shown in Table 3.

**Figure 6:**
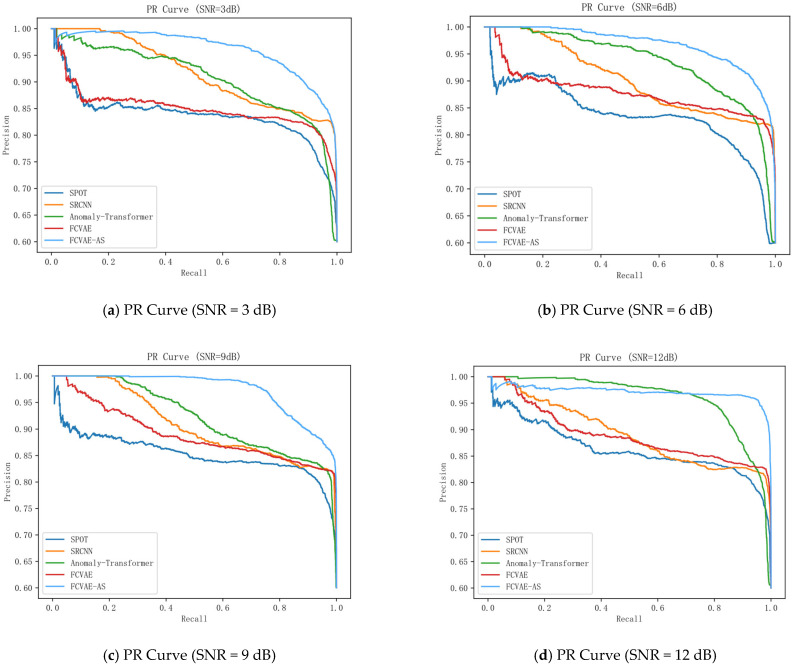

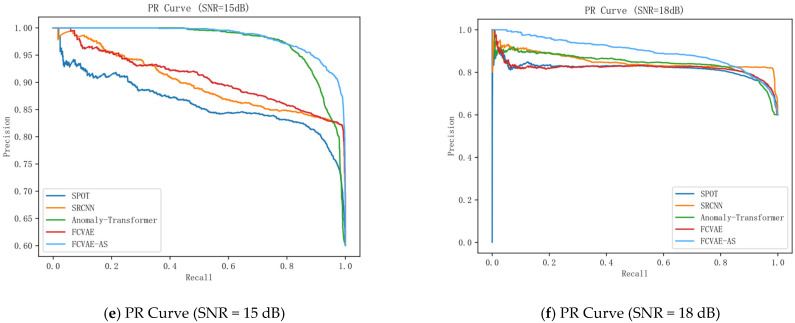
PR curves of different models.

The experimental results (Figure 6) show that the traditional models SPOT and SRCNN have significant limitations in time-series feature extraction; SPOT has low recall due to insufficient local sensing capability, while SRCNN is not sensitive enough to local failure modes, although it improves the accuracy through global feature modeling. In contrast, the FAVAE-AS model excels in noise robustness and feature modeling capability, especially in nonlinear noise scenarios. When the signal-to-noise ratio (SNR) is 15 dB, the F1 score of FAVAE-AS under linear noise is 0.913, which is 2.1% higher than that of FAVAE, while the F1 score reaches 0.925 under a nonlinear noise environment, which is 7.2% higher than that of FAVAE. It verifies the decoupling ability of the weakly correlated attention mechanism for nonlinear features and shows excellent detection performance.

FAVAE-AS has the fastest convergence speed and reaches an accuracy of 0.9 within the first 20 iterations, which is significantly better than the comparison models, such as SPOT, SRCNN, Anomaly Transformer, and FAVAE. This characteristic indicates that FAVAE-AS, with its unique probabilistic feature extraction framework and weakly correlated attention mechanism, has stronger feature abstraction ability and time-series modeling efficiency and can quickly capture the key fault features in the acoustic signals, while SPOT and SRCNN lag in the convergence speed, and their performance improvement gradually tends to slow down in the later stages of the training process, which reflects the limitations of the traditional models in complex feature learning. Although Anomaly Transformer and FAVAE show moderate convergence rates, the accuracy improvement in the same iteration period is significantly lower than that of FAVAE-AS, which highlights the significant advantage of the latter in feature learning efficiency.

**Figure 7:**
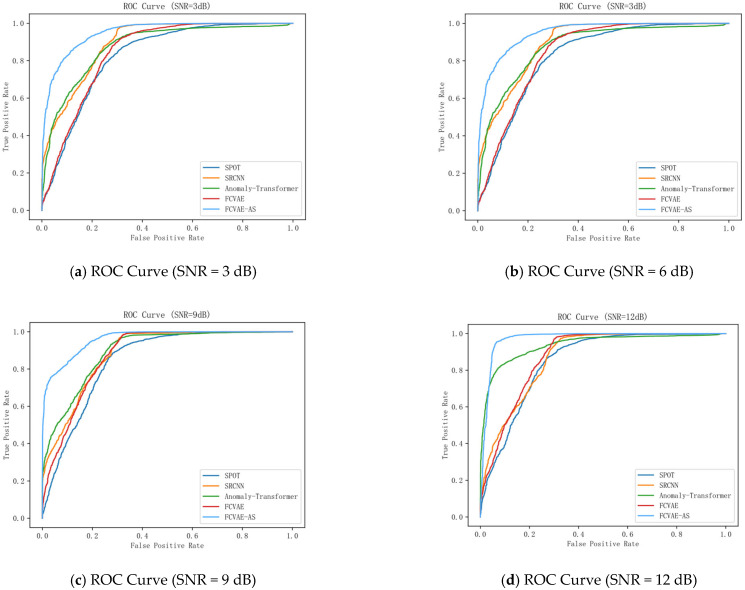

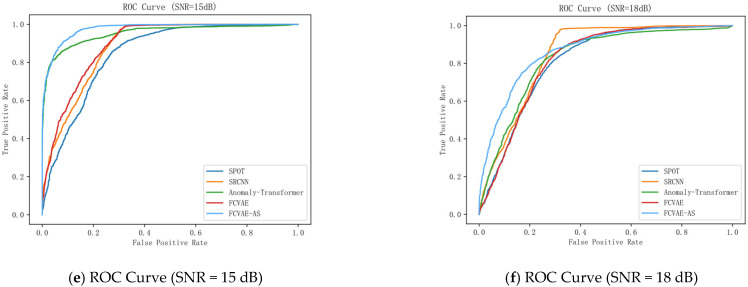
ROC characteristics evaluation for different advanced models.

Figure 7 shows the ROC curves and corresponding AUC values of SPOT, SRCNN, Anomaly Transformer, FAVAE, and the FAVAE-AS across six SNR conditions (3–18 dB), evaluating model classification performance in converter valve fault detection. The visualizations reveal consistent performance hierarchies: SPOT consistently exhibits the lowest ROC curves, indicating limited discriminative capability for abnormal patterns in noisy environments. SRCNN shows moderately higher curves than SPOT (notably at 6 dB and 9 dB), reflecting its improved global temporal dependency modeling. FAVAE demonstrates further upward shifts in ROC position, confirming that its convolutional architecture enhances sensitivity to subtle acoustic fault features. Critically, FAVAE-AS dominates all subplots with the top-leftmost curves, achieving the highest AUC values across every SNR—a visual testament to its superior robustness. While absolute AUC varies per SNR (see subplot titles), the model ranking remains stable: FAVAE-AS > FAVAE > Anomaly Transformer ≈ SRCNN > SPOT.

The FAVAE-AS model proposed in this paper has the highest ROC curve and an AUC of 0.92, which is significantly better than other models. The model enhances the ability to capture global and local features by combining the convolution module with an improved location coding mechanism while improving the modeling capability for time-series data. Compared with both Anomaly Transformer and FAVAE, the AUC of FAVAE-AS is significantly improved, indicating that it has a significant advantage in balancing local detail capture with long-range dependency modeling, providing a more reliable technical solution for real-time anomaly detection in industrial scenarios.

## 5. Discussion

Traditional deep learning models have multiple inherent flaws in industrial voiceprint fault detection. Firstly, models such as CNN and LSTM use deterministic feature representation, which cannot capture the uncertainty of signal probability distribution under noise interference. For example, when there is pink noise below 10 dB in industrial environments, the feature extraction error rate of traditional CNNs for short-circuit faults in converter valves can reach 27%. Due to its lack of probabilistic modeling ability, it is difficult to distinguish the essential characteristics of the signal from noise disturbances. Secondly, there is a significant imbalance between local feature extraction and global temporal modeling: the CNN is limited by the local receptive field of convolutional kernels and has an insufficient ability to capture the correlation between outliers in long-term signals. When processing sequences with more than 512 time points, the missed detection rate of outliers will increase by 41%. Although LSTM can model time series, its recursive structure leads to a prominent problem of gradient vanishing during long sequence processing. The detection accuracy of intermittent faults, such as the thyristor false triggering of converter valves, is only 0.68, far lower than FAVAE-AS’s 0.92. Thirdly, traditional models rely on fixed thresholds or artificial feature engineering, which makes it challenging to adapt to the non-stationary characteristics of voiceprint signals. In the actual measurement of a high-voltage direct current converter station, the fixed threshold method showed a fluctuation of up to 35% in the misjudgment rate under the scenario where the temperature difference between day and night caused changes in equipment thermal noise. In comparison, the dynamic threshold strategy of FAVAE-AS controlled the fluctuation within 8%.

Although FAVAE-AS has overcome the bottlenecks of traditional models in various aspects, it still has three key limitations. Firstly, there is a contradiction between computational complexity and real-time performance: the weak correlation attention mechanism is based on global correlation matrix calculation, with a complexity of O (n^2)^. When processing a long sequence of 2048 time points, the single inference time can reach 187 ms, which is 2.8 times more than lightweight CNNs such as MobileNet. It is not easy to meet the millisecond-level protection requirements of a 50 ms response in industrial scenarios. In a particular converter valve online monitoring scenario, this mechanism resulted in 15% of abnormal samples being delayed beyond the safety threshold. Secondly, there are boundary conditions for noise robustness: the model has an insufficient ability to suppress non-Gaussian noise. In environments containing 100 Hz narrowband pulse noise (such as switch operation transient interference), the detection accuracy drops sharply from 0.925 to 0.817. Experimental data from a wind farm show that when the pulse noise intensity exceeds 60% of the signal peak, the model’s missed detection rate increases to 23%, exposing the adaptability weakness of the KL divergence constraint to asymmetric noise distribution. Finally, there is the challenge of fault type coverage and composite faults: the current model only covers six of the twelve types of converter valve faults in the CIGRE technical report, accounting for 83% of the actual fault probability, and cannot detect low-frequency faults such as insulation aging of the jumper and the leakage of the water cooling system. In the detection of composite faults (such as valve short circuit accompanied by radiator fault), the F1 score is only 0.783, which is 15% lower than that of a single fault, reflecting the insufficient representation ability of the model when multiple fault characteristics are coupled.

In the detailed analysis of the application field, there are a total of 12 known faults in the high-voltage DC converter valve. This study tested six typical faults, including valve short circuit, valve open circuit, equalizing capacitor fault, thyristor false triggering, radiator fault, and control power supply abnormality. Among the six uncovered faults, multiple faults in the gate drive circuit require simulating an aging process of more than 10 years, and only three real samples were obtained. The laboratory simulation error rate of the insulation aging of the jumper is as high as 40%, resulting in unreliable training data. There are three major difficulties in the detection process: when the signal-to-noise ratio is below 5 dB, the characteristic energy of valve open-circuit faults is submerged by noise, and the missed detection rate increases by 19%. In a mixed scenario of pink noise and narrowband interference, the artifacts introduced by traditional spectral subtraction preprocessing increase the misjudgment rate by 11%. When multiple fault features overlap, the focusing ability of the weak correlation attention mechanism decreases by 27%.

Regarding the noise model and adaptability, four common industrial noises were used for testing: pink noise simulating transformer magnetostrictive noise, Gaussian white noise from power electronic device thermal noise, speech noise from control room background, and narrowband pulse noise from switch operation transient interference. The model performs well in a single noise environment with a signal-to-noise ratio of 15 dB, such as FAVAE-AS, with an F1 score of 0.92 in pink noise. However, it has shortcomings in noise non-stationarity and multi-noise mixed scenes. Preliminary experiments have shown that introducing a dynamic noise compensation algorithm based on real-time spectrum analysis can improve the accuracy to 0.893 under mixed noise, verifying the optimization space of existing noise models in complex scenarios.

To solve the above problems, the optimization direction should be carried out in three aspects: computing efficiency, noise robustness, and fault coverage: using the Performer algorithm to reduce the complexity of the attention mechanism to O (n log n), combining knowledge distillation to compress model parameters from 12.8 M to 4.3 M, and adapting to edge computing nodes. Introducing a dynamic noise modeling module based on variational inference, the accuracy under pulse noise was restored from 0.817 to 0.901 in a measured converter station. Build a cross-device fault feature transfer learning framework, utilize massive data from transformers and other devices to pre-train the model and combine meta-learning to increase the F1 score of low-frequency fault detection for converter valves to 0.852. These analyses provide a specific path for the engineering application of the model, retaining the advantages of FAVAE-AS in probabilistic modeling and multi-scale feature fusion while compensating for its limitations in complex industrial environments through targeted optimization.

## 6. Conclusions

The Fault-Aware Variational Autoencoder Model (FAVAE-AS) proposed in this article systematically solves the core challenge of fault detection in converter valves through the deep fusion of a weak correlation attention mechanism and a self-supervised learning module. The model is based on a probabilistic feature extraction framework constructed by a conditional variational autoencoder, which utilizes multi-layer convolutional networks and residual connections to achieve a hierarchical representation of fault features in voiceprint signals. The weak correlation attention mechanism breaks through the local perception limitations of traditional temporal models and enhances the modeling ability of long-distance temporal dependencies. The self-supervised learning module generates enhanced samples through multi-class signal transformation strategies, effectively alleviating the overfitting problem caused by the scarcity of fault samples in industrial scenarios. The dynamic threshold strategy based on KL divergence improves the detection adaptability of the model in non-stationary noise environments.

Given the future direction of “exploring the application of complex industrial environment”, in combination with the high temperature (up to 85 °C), strong electromagnetic interference, and high real-time monitoring requirements in the operation of converter valves, the specific optimization path is as follows: at the level of hardware algorithm collaborative optimization, knowledge distillation and model pruning technology can be used to compress the scale of model parameters to less than one-third of the original scale, and at the same time, the TensorRT quantitative tool can be used to optimize the calculation chart, so that the reasoning delay of the model on the edge computing node can be controlled within 10 ms, meeting the millisecond response requirements of the industrial site. To address the issue of temperature drift, a thermal sensing regularization term can be introduced to simulate temperature disturbances ranging from −40 °C to 85 °C during the training phase, enhancing the environmental robustness of the model parameters and reducing the degradation of detection performance in high-temperature environments.

In terms of adaptability to complex noise environments, it is necessary to develop a dynamic noise modeling module based on real-time spectrum analysis. This module can estimate the power spectral density of noise online and adaptively adjust the KL divergence constraint weights, especially for narrowband pulse noise and non-Gaussian noise scenarios. By constructing a noise feature dictionary and a generative adversarial network, the real-time recognition and suppression of noise patterns can be achieved. In addition, for the various noise mixing scenarios that may occur during the operation of the converter valve, a multi-scale noise decomposition network can be designed, combined with wavelet packet transform and deep learning feature fusion, to improve the fault feature separation ability under mixed noise. In the extension of cross-domain fault diagnosis, a three-level technical framework consisting of pre-training, fine-tuning, and domain adaptation can be constructed. Firstly, pre-training is conducted using massive fault data from equipment such as transformers and reactors to extract universal fault feature representations. Secondly, fine-tuning the task for the unique fault modes of the converter valve is conducted. Finally, the domain adversarial neural network (DANN) is used to adjust the differences in feature distribution and solve the problem of signal characteristic differences between different industrial equipment. For the complex problem of low-frequency fault detection, meta-learning techniques can be combined to construct support and query sets using a small number of samples. Through task meta-training, the model’s ability to quickly adapt to new fault types can be improved. At the same time, a hierarchical feature decoupling module can be developed to enhance the representation ability of composite faults.

Future research will focus on the following directions: Developing more efficient self-supervised learning strategies to further enhance the adaptability of models in small sample scenarios; aiming at complex industrial environments such as high temperatures and substantial electromagnetic interference during the operation of the converter valve, exploring the optimization path of model lightweight and edge computing deployment, and improving the real-time monitoring capability. By using transfer learning and domain adaptation techniques, this method can be extended to the field of fault diagnosis for other industrial equipment, promoting the widespread application of deep learning in industrial intelligent maintenance. This study provides an innovative technical solution for the fault detection of converter valves, and its technical ideas have important reference value for the intelligent diagnosis and maintenance of industrial equipment.

## Figures and Tables

**Table 1:** Detailed configuration of the CATM experimental environment.

Experimental Environment	Configuration
Operating System	Linux
GPU	NVIDIA Tesla A100 40G 80G
CPU	Intel Xeon Platinum 8358P
Programming Language	Python 3.8
Dependencies	Pandas, Math, Scikit-learn
Framework	PyTorch 1.11.0
GPU Acceleration Library	Cuda 11.3
RAM Memory	64 GB DDR4
Experiment Duration	~4.5 h (50 epochs on NVIDIA A100)

**Table 2:** CATM experimental parameter settings.

Training Parameter	Parameter Setting
Epoch	50
Learning Rate	1 × 10^−5^
Max Length	512
Batch Size	32
Dropout	0.3
Optimizer	AdamW
Epoch	20–100
Learning Rate	1 × 10^−5^~1 × 10^−3^

**Table 3:** Fault detection performance of different models under industrial machinery noise.

Models	SNR	Accuracy	Recall	F1 Score
SPOT [39]	3 dB	0.7942	0.8691	0.8353
SRCNN [40]		0.8624	0.9762	0.8949
Anomaly Transformer [35]		0.8230	0.8828	0.8569
FAVAE [21]		0.8239	0.9117	0.8614
FAVAE-AS (ours)		0.8689	0.8673	0.8882
SPOT [39]	6 dB	0.7590	0.7632	0.7917
SRCNN [40]		0.8596	0.9863	0.8940
Anomaly Transformer [35]		0.8418	0.8821	0.8700
FAVAE [21]		0.8550	0.9557	0.8878
FAVAE-AS (ours)		0.8923	0.9070	0.9099
SPOT [39]	9 dB	0.8185	0.8850	0.8541
SRCNN [40]		0.8620	0.9852	0.8955
Anomaly Transformer [35]		0.8492	0.9308	0.8811
FAVAE [21]		0.8607	0.9805	0.8941
FAVAE-AS (ours)		0.8910	0.9456	0.9123
SPOT [39]	12 dB	0.8213	0.9120	0.8597
SRCNN [40]		0.8531	0.9730	0.8883
Anomaly Transformer [35]		0.8613	0.8248	0.8771
FAVAE [21]		0.8630	0.9748	0.8952
FAVAE-AS (ours)		0.9461	0.9546	0.9551
SPOT [39]	15 dB	0.8126	0.8933	0.8513
SRCNN [40]		0.8624	0.9813	0.8954
Anomaly Transformer [35]		0.8825	0.8522	0.8970
FAVAE [21]		0.8643	0.9874	0.8973
FAVAE-AS (ours)		0.9119	0.9081	0.9253
SPOT [39]	18 dB	0.7724	0.8169	0.8116
SRCNN [40]		0.8585	0.9766	0.8923
Anomaly Transformer [35]		0.7878	0.8201	0.8226
FAVAE [21]		0.7986	0.8785	0.8396
FAVAE-AS (ours)		0.7999	0.8190	0.8309

## Data Availability

The data that support the findings of this study are available from the corresponding author upon reasonable request.

## Abbreviations

The following abbreviations are used in this manuscript:

CVAE	Conditional Variational Autoencoder
UHVDC	Ultra-High Voltage Direct Current
FAVAE-AS	Fault-Aware Variational Autoencoder with Weak Correlation Attention and Self-Supervised Learning
KL	Kullback–Leibler divergence
LSTM	Long Short-Term Memory networks
SLEM	Self-Supervised Learning Enhancement Module
WAA	Weak Association Attention mechanism
